# The Italian version of the Female Genital Self-Image Scale: psychometric properties and associations with sexual function and psychological health

**DOI:** 10.1186/s40359-026-04030-6

**Published:** 2026-01-28

**Authors:** Federica Facchin, Andrea Bonanomi, Denise Vagnini, Giulia Emily Cetera, Emanuela Saita

**Affiliations:** 1 Department of Psychology, Università Cattolica del Sacro Cuore, Milan, Italy; 2 Department of Statistical Science, Università Cattolica del Sacro Cuore, Milan, Italy; 3 Gemelli Women Health Center for Digital and Personalized Medicine, Dipartimento Scienze della Vita e Sanità Pubblica, Sezione di Ginecologia e Ostetricia, Università Cattolica del Sacro Cuore, Rome, Italy

**Keywords:** Body appreciation, Female genital Self-Image scale, Italian translation, Psychological wellbeing, Psychometric properties, Self-esteem, Sexual function

## Abstract

**Background:**

Since its initial development, the Female Genital Self-Image Scale (FGSIS) has been translated into various languages, including Arabic, Turkish, Persian, Thai, Spanish, and Swedish, but no Italian translations are currently available. The aim of this study was to examine the psychometric properties of the first Italian translation of the FGSIS, named FGSIS-It—including its factor structure, internal consistency, and convergent validity—and to explore its associations with female sexual function and psychological health.

**Methods:**

The study employed a cross-sectional design, and data were collected online using Qualtrics. A snowball sampling approach was employed, recruiting participants through social media and educational platforms. Participants were 432 individuals assigned female at birth, aged over 18 years, who completed all sections of the FGSIS-It. In addition to sociodemographic data, information was collected on sexual function using the Female Sexual Function Index, and on psychological health using the Body Appreciation Scale-2, the Rosenberg Self-Esteem Scale, and the Psychological General Wellbeing Index-Short. After examining the psychometric properties of the FGSIS-It through both Exploratory and Confirmatory Factor Analyses, as well as Cronbach’s *α*, McDonald’s *ω*, and Pearson correlations, we investigated its associations with female sexual function and psychological health.

**Results:**

Exploratory factor analysis and confirmatory factor analyses supported a one-factor structure. The scale demonstrated good internal consistency (Cronbach’s *α* = 0.811; McDonald’s *ω* = 0.823). Significant positive correlations, ranging from weak to moderate, were found between female genital self-image and both female sexual function and psychological health. A poorer female genital self-image was observed in individuals aged < 45 years who reported a risk of sexual dysfunction, compared to those without such risk, based on the FSFI total score.

**Conclusion:**

The FGSIS-It is a reliable instrument for assessing female genital self-image among Italian individuals assigned female at birth, drawn from the general population.

**Supplementary Information:**

The online version contains supplementary material available at 10.1186/s40359-026-04030-6.

## Background

### Female genital self-image: theoretical framework

Female genital self-image refers to a person’s subjective perception of their genitalia, encompassing associated attitudes, thoughts, feelings, and behaviors [1, 2]. Over the past ten years, a growing number of studies have emphasized the importance of female genital self-image in relation to the sexual and psychological health of individuals assigned female at birth, although the overall body of research remains limited. In a recent article, Mohammed et al. [1] proposed a psychological and sociocultural perspective for understanding female genital self-image, situating this construct within a theoretical framework that encompasses body image and sexual self-concept theories. Female genital self-image has been conceptualized as an important component of overall body image, shaped by the complex interplay of psychological and sociocultural factors, as well as social and sexual experiences [3].

Investigating genital self-image, especially female genital self-image, is relevant in contemporary society. Today, being dissatisfied with the appearance of one’s own genitals is not uncommon. In Western culture, female genitalia are often idealized as hairless, symmetrical, and minimally protruding, a standard sometimes referred to as the “Barbie Doll” vulva [4]. Despite the considerable anatomical diversity within the normal range of female genitalia, some individuals may feel compelled to conform to societal ideals, sometimes opting for non-medically indicated cosmetic procedures [5]. Media, social networks, online pornography, and societal trends, such as pubic hair removal, have reinforced this “idealized” representation of vulvar appearance [6, 7]. Female genital self-image can also be influenced by family, peers, and friends, and may not benefit from a form of sex education that focuses primarily on preventing sexually transmitted diseases (which is indeed important), rather than fostering body—and specifically genital—awareness [1].

Poor female genital self-image can significantly affect multiple health domains. Individuals dissatisfied with their genitals are more likely to delay or avoid medical visits, thereby missing opportunities for preventive care [8]. Moreover, research has linked individuals’ perceptions and feelings about their genitals to sexual experiences since the 1980s [9], suggesting that genital self-image is an important aspect of sexual health. Individuals with a positive genital self-image tend to feel more sexually attractive and less inhibited about their body, and report better sexual functioning, greater satisfaction and quality of intimate experiences, and higher self-esteem [1, 10].

### Female genital self-image in the Italian context

There is a very limited body of research examining female genital self-image in the Italian context. In a study by DeMaria et al. [11], 46 Italian women aged 19–45 were interviewed about their perceptions of their genitals. Participants reported that discussions about genitals are considered highly private and often uncomfortable, occurring mainly in health-related contexts. Many women expressed embarrassment or reluctance to talk about their genitals, even with friends, highlighting a cultural tendency to avoid open conversation and a general lack of awareness. Partners and female friends were important sources of influence, shaping perceptions of genital acceptability.

Women often reported concerns about genital odor, consistent with findings from other cultural contexts [12], which led to rigorous hygiene practices such as frequent use of the bidet, cleansing routines taught by mothers, and extra care during menstruation. Attitudes toward genital appearance were often neutral to positive, with many women seeing their genitals as just another part of the body. Perceptions generally improved with age, shifting from early negative feelings to greater acceptance and a sense of normalcy. Positive perceptions were linked to comfort, satisfaction, a sense of ownership, and attention to genital care and overall wellbeing. Cosmetic genital surgery was largely rejected, with women valuing natural appearance and associating genital health with sexual pleasure and relationship satisfaction, often influenced by long-term partnerships and sexual experiences.

In another study conducted by Preti et al. [13], among 500 participants, only 15% were able to accurately sketch vulvar anatomy. The majority (76%) had never heard of vulvar self-examination, and 61% reported feelings of shame or embarrassment toward their genitals. Only 23% stated they would seek medical advice if they noticed potential abnormalities during a self-exam. Nevertheless, most participants (69%) expressed strong interest in receiving more information about vulvar self-examination and vulvar health through educational materials such as videos or social media.

Taken together, these findings suggest that female genital self-image remains a largely neglected concept in Italy, shaped by cultural taboos, medicalization, and the scarcity of assessment tools. In this context, a validated instrument such as the Female Genital Self-Image Scale (FGSIS) could be particularly useful for both research and clinical practice.

### Development of the female genital self-image scale

In 2010, Herbenick and Reece presented, for the first time, a 7-item self-report questionnaire, the Female Genital Self-Image Scale (FGSIS), aimed at assessing how people assigned female at birth perceive and experience their external genitalia, focusing on appearance, odor, and function [2]. The authors aimed to provide a new tool for examining female genital self-image using a rational-empirical, comprehensive, and contemporary approach, considering the overlooked nature of such a sensitive psychological construct and the lack of questionnaires with clear evidence of their psychometric properties [2, 14].

The two-phase study conducted by Herbenick and Reece [2] involved the online administration of the FGSIS to a convenience sample of 1,937 women (mean age = 29.55 years). Participants also completed a researcher-developed questionnaire designed to collect demographic information and data related to sexual and health behaviors, as well as the Female Sexual Function Index (FSFI; [15]). The psychometric properties of the FGSIS were assessed through analyses of internal consistency (Cronbach’s alpha) and construct validity (factor analysis using principal component extraction). Results supported a one-factor structure, accounting for 59.23% of the variance. Predictive validity was evidenced by significantly higher FGSIS scores among participants who reported experiencing orgasm through cunnilingus or vibrator-assisted self-masturbation, or who had undergone a gynecological exam within the past year, compared to those who had not. Additionally, the FGSIS scores showed significant correlations with female sexual function as measured by the FSFI.

In a second study [14], the psychometric properties of the FGSIS, including its temporal stability, were reexamined among a nationally representative sample of 3,800 women aged 18–60 years, and confirmatory factor analysis was used to evaluate its model of fit. Following these analyses, the authors removed three items from the original FGSIS and proposed a 4-item version (the FGSIS-4) that showed good construct validity, internal reliability, temporal stability, and predictive validity. Specifically, better genital self-image was associated with masturbation, positive health behaviors (including seeking gynecological exams and performing genital self-examinations), and greater sexual function. The authors concluded that, although the FGSIS-4 was found to be a better fit than the FGSIS-7, both scales may be useful depending on the researchers’ needs and aims.

In a subsequent study [8], principal component analysis revealed a two-factor solution, explaining 72.59% of the variance. Factor 1 was named “Intrapersonal Concerns” and Factor 2 “Interpersonal Concerns”. Confirmatory factor analysis indicated that the two-factor solution fitted the data better compared to the original one-factor solution. The authors concluded that a two-factor solution may reflect the multifactorial nature of genital self-image, capturing the distinction between intrapersonal and interpersonal concerns. However, they encouraged further research to examine the psychometric properties of the FGSIS in a more geographically diverse population.

### Available validations of the FGSIS

Since the initial development of the FGSIS, the 7-item version has been the most widely used questionnaire in a variety of studies. The scale has been translated and adapted into several languages, including Persian [16], Arabic ( [17]; 4-item version), Turkish [18], Thai [19], Swedish [20], and Spanish [21]. In these studies, with sample sizes ranging from 86 [19] to 1,877 participants [16], the FGSIS showed satisfactory internal consistency, with Cronbach’s alpha values ranging from 0.82 [18, 20] to 0.97 [17]. The main findings from this body of validated translations—focusing on the FGSIS factor structure and the scale version used (7-item or 4-item)—are summarized in Supplemental Table 1, along with results from the original validation studies [2, 14]. Overall, the translation studies revealed mixed findings, suggesting that the FGSIS is characterized by flexibility in its factor structure [18, 19]. Significant correlations were identified between the FGSIS and other constructs, including sexual function, body appreciation, and self-esteem [16–18].

### The current study

The aims of this study were: (i) to provide an Italian translation of the FGSIS (the FGSIS-It), developed by a team of expert clinicians; and (ii) to examine the psychometric and structural properties of the FGSIS-It in a sample of individuals assigned female at birth from the general population, using both Exploratory Factor Analysis (EFA) and Confirmatory Factor Analysis (CFA) to evaluate the goodness of fit of the EFA-derived solution; and (iii) to further investigate the associations between the construct of interest—female genital self-image as measured by the FGSIS-It—and female sexual function, body appreciation, self-esteem, and psychological wellbeing. We chose the 7-item version of the FGSIS rather than the 4-item version to allow comparisons with previous translations, noting that only the Arabic translation [17] was based on the shorter version. Moreover, given the scarcity of Italian studies on female genital self-image and the limited availability of assessment instruments, we opted to begin by using the original 7-item scale as a first step toward systematically exploring this construct in the Italian context.

Drawing on the studies summarized in Supplemental Table 1, we expected to find a one-factor structure for the FGSIS-It using exploratory factor analysis; however, considering the structural flexibility reported in the published literature [2, 8, 14, 16–21], we also compared the one-factor and two-factor solutions. In line with previous translation studies, we anticipated that the FGSIS-It would show good internal consistency (Cronbach’s alpha > 0.80) and positive associations with female sexual function, body appreciation, and psychological wellbeing.

## Methods

The findings reported in this article were derived from the first part (Phase 1) of a two-step online research focused on female genital self-image, psychological wellbeing, sexual function, and health-related behavior. The specific aim of the first part of this larger research was to examine the psychometric properties of the first Italian translation of the Female Genital Self-Image Scale. Permission to translate and use the scale was kindly given by Debra Herbenick on May 26, 2022. Ethical approval was granted by the Ethical Commission for Research in Psychology, Department of Psychology, Catholic University of the Sacred Heart (protocol number: 57 − 23; approval date: May 24, 2023).

This study included individuals assigned female at birth, irrespective of their gender identity or sexual orientation, aged > 18 years. Participants who did not complete the FGSIS-It in all its parts were excluded. Based on these criteria, the final sample was composed of 432 participants. Data were collected online from June 2023 to April 2025 using Qualtrics (Qualtrics Ltd.). Participants were recruited using a snowball sampling approach, which involved posting the invitation to participate in the study on social media (Facebook, Instagram) and Blackboard Learn, an application for online teaching and learning provided by the institution of the first author. Data collection was carried out anonymously, and participants provided electronic informed consent (without identifying information) before completing the survey.

### Italian translation of the female genital self-image scale (FGSIS-It)

The FGSIS is composed of 7 items evaluating people’s feelings and beliefs about their own female external genitalia. Responses are rated on a 4-point Likert scale (from 1 = strongly disagree to 4 = strongly agree). A total score is calculated as the sum of the 7 items, with values ranging from 7 to 28. Higher scores denote a more positive genital self-image [8].

Following the work of other authors [22], we used a forward- and back-translation approach. The original FGSIS was translated from English into Italian (forward translation) by the first author. A back translation was subsequently performed by the fourth author, a bilingual gynecologist, and reviewed by the research team. To qualitatively assess *face validity* (defined as the extent to which the questionnaire items appear relevant, accurate, and understandable based on the subjective judgment of experts or members of the target population) and *content validity* (defined as the ability of the questionnaire to measure the construct of interest), the final version of the scale (named FGSIS-It) was reviewed by a panel of experts not involved in the study, including two gynecologists, a midwife, two psychologists, and a clinical sexologist, who provided qualitative verbal feedback. None of the experts raised significant concerns regarding the Italian translation, and all seven items from the original version were retained. The final version of the FGSIS-It is provided as supplemental material (see Supplemental Table 2).

### Other measures

#### Sociodemographic data

A set of researcher-developed questions was used to collect information on participants’ age, relationship status (including whether they currently had a sexual partner and the partner’s gender), education level, employment status, current pregnancy, number of children, history of vaginal deliveries, and presence of gynecological conditions.

#### Sexual function

The Female Sexual Function Index (FSFI; [15]; Italian validation by Filocamo et al. [23]) was administered to assess sexual function, only to participants who reported having a current sexual partner. This 19-item questionnaire evaluates six domains of sexual function: desire, arousal, lubrication, orgasm, pain, and satisfaction. The total FSFI score ranges from 2 to 36, with higher scores reflecting better sexual functioning. A clinical cutoff score of 26.55 has been established to distinguish premenopausal women at risk of sexual dysfunction from those not at risk [24, 25]. In the present study, Cronbach’s *α* coefficients ranged from 0.86 for the Satisfaction domain to 0.97 for Pain and total FSFI score.

#### Body appreciation

We measured body appreciation using the Italian validated version of the Body Appreciation Scale-2 (BAS-2; [22, 26]), composed of 10 items (e.g., “I respect my body”, “I feel good about my body”) rated on a 5-point Likert scale from 1 = *Never* to 5 = *Always*, with higher total scores (ranging from 5 to 50) indicating greater body appreciation. In our study, the BAS-2 showed very good internal consistency, with Cronbach’s *α* of 0.93.

#### Self-esteem

The Rosenberg Self-Esteem Scale (RSES; [27]; Italian version validated by Prezza et al. [28]) was used to assess participants’ global self-worth and overall feelings about themselves. The scale consists of 10 items rated on a 4-point Likert scale (0 = Strongly Disagree, 3 = Strongly Agree), with higher scores indicating greater self-esteem. For the RSES, the value of Cronbach’s *α* was 0.91.

#### Psychological wellbeing

Psychological wellbeing was measured using the Psychological General Well-being Index-Short (PGWB-S; Grossi et al. [29]), a brief 6-item instrument employing a 6-point Likert scale. Total scores range from 6, indicating the lowest level of wellbeing, to 36, representing the highest. In the present study, the PGWB-S demonstrated good internal consistency, with a Cronbach’s *α* of 0.85.

### Data analysis

Statistical analyses were performed using IBM SPSS 29 and Jamovi 2.6.23. Descriptive statistics were computed for all variables to present the overall characteristics of the study population. Skewness and kurtosis values between − 2 and + 2 were deemed acceptable indicators of normal distribution [30, 31].

The psychometric properties of the FGSIS-It were evaluated following the procedures used in previous translation studies [18, 19]. Descriptive statistics were calculated for each item, including the mean, standard deviation, skewness, and kurtosis.

The sample was then randomly divided into two subsamples. Preliminary analyses, including independent samples *t*-tests and Chi-square tests where appropriate, were conducted to assess whether the two subgroups differed on any of the study variables. No significant differences were observed (all *p* values > 0.05). The structural properties of the FGSIS-It were examined using Exploratory Factor Analysis (EFA) on data from the first subgroup (*N* = 201, representing 46.5% of the total sample). As done in previous research [31], we chose the principal axis method for factor extraction with Promax rotation. The adequacy of the sample and the suitability of the data for factor analysis were assessed using the Kaiser–Meyer–Olkin (KMO) measure, Bartlett’s test of sphericity, and the examination of factor loadings. To assess *internal consistency*, Cronbach’s *α* coefficients were calculated, as in all published studies examining the psychometric properties of the FGSIS—both the original version and the available translations. Additionally, since one study [21] reported only McDonald’s *ω*, this coefficient was also computed to allow for comparisons with all published translations. In addition, item–total Pearson correlations were calculated to assess whether all items contributed significantly to the same latent construct [19].

Subsequently, data from the second subsample (*N* = 231, representing 53.5% of the total sample) were analyzed using Confirmatory Factor Analysis (CFA) in Jamovi 2.6.23. Due to the ordinal nature of the items, the model was estimated using the Diagonally Weighted Least Squares (DWLS) method with robust standard errors, and assessed through approximate fit indices, following the recommendations of Hu and Bentler [32]. In this set of analyses, we compared the one-factor and two-factor solutions to determine which provided the best fit for our data. The fit indices considered were: a Root Mean Square Error of Approximation (RMSEA) below 0.08, a Confirmatory Fit Index (CFI) exceeding 0.95, and a Tucker-Lewis Index (TLI) greater than 0.90 [32]. To improve the structure of the model, modification indices were considered.

To further examine the psychometric properties of the FGSIS-It scale using a complementary analytical framework, a Partial Credit Rasch Model (PCM) was applied. This model was selected because it is appropriate for ordinal items that may show different step difficulties across items (i.e., unequal distances between adjacent response categories). The parameters of the Rasch model were estimated using the Maximum Likelihood (ML) method. Model reliability was evaluated through the Person Separation Index (PSI), which reflects the ability of the scale to discriminate between respondents with different levels of the latent trait. PSI values above 0.80 are generally considered acceptable, indicating good reproducibility of person estimates [33]. To assess item fit to the Rasch model, Infit and Outfit mean square (MNSQ) statistics were computed; acceptable values typically range between 0.6 and 1.4, suggesting adequate model-data fit [34]. Finally, item difficulty and step threshold parameters were inspected to verify the proper ordering of response categories and to confirm the monotonic progression of the scale.

In the final set of analyses, conducted on the total sample, associations between the FGSIS-It and other standardized questionnaires included in the study (FSFI, BAS-2, RSES, PGWB-S) were examined using Pearson correlation coefficients. These analyses also aimed to assess *convergent validity*, defined as the extent to which a measure is positively correlated with other instruments that assess similar or theoretically related constructs [35]. In a previous study aimed at validating the FGSIS in Iran [16], Pakpour and colleagues employed the FSFI, RSES, and BAS to assess convergent validity. These authors found significant positive correlations between the FGSIS-I and the FSFI (*r* values ranging from 0.18 for lubrication to 0.32 for the FSFI total score), the RSES (*r =* 0.41), and the BAS (*r =* 0.49). In our study, we also included the PGWB-S because of the available evidence regarding the association between genital body image and psychological wellbeing [5].

To further investigate the relationship between female genital self-image and sexual function, we compared the subgroup of participants at higher risk of experiencing sexual dysfunction to those not at risk, using analysis of variance (ANOVA) and controlling for potential confounders (age and presence of gynecological conditions). Because the FSFI cut-off score of 26.55 was established considering premenopausal women, we excluded from this analysis participants aged ≥ 45 years (only 31, 7% of the whole sample). For this analysis, we report the effect size as partial *η²*. According to Cohen [36], partial *η²* values of approximately 0.01 indicate small effects, values around 0.06 indicate medium effects, and values of 0.14 or greater indicate large effects.

The association between female genital self-image and sexual function, measured using the FSFI, was addressed in the validation study of the FGSIS conducted by Herbenick and Reece [2], with significant but weak positive correlations (except for the FSFI Desire subscale), with *r* values ranging from 0.13 for the Pain domain to 0.20 for the overall FSFI score. To interpret the Pearson correlations performed in our study, the following ranges of *r* values were considered: 0.10–0.20 indicate weak correlations, 0.20–0.50 indicate moderate correlations, and values greater than 0.50 indicate strong correlations [36].

Findings were considered statistically significant if *p* values were < 0.05. Sample size was calculated a priori based on the following criteria: (i) when the objective of a study is the validation of a scale, the general rule is to include 10–20 participants per item [37]. The FGSIS consists of 7 items; (ii) in validation studies of quantitative instruments that employ procedures such as CFA, it is advisable to determine the minimum sample size as a range, tailoring it according to the specifics of the study and the target population, while also considering previously conducted validation studies on the same instrument [37, 38]; (iii) in validation studies targeting the general population, such as the one proposed in this phase, it is recommended to recruit between 375 and 500 participants [37]; (iv) to obtain stable correlations in observational studies, a minimum of 250 participants is required [39]. For the reasons outlined above, a sample size ranging from 375 (minimum) to 500 (maximum) participants was determined for this study. For the FGSIS-It, there were no missing data, as only participants who completed the entire questionnaire were included. For all other questionnaires, missing data were not imputed, and the exact number of respondents is reported in Table 1.

**Table 1:** Characteristics of the study population

Sociodemographic data (*N *= 432)		
Age in years (M ± SD)		26.14 ± 9.26
Relationship status (*N *[%])	In a relationship	250 (58)
	Married	38 (9)
	Separated/divorced	/
	Single	144 (33)
Sexual partner (*N *[%])	Yes	307 (71)
	No	125 (29)
Gender of partner (*N *[%]) (if sexual partner = yes)	Male	283 (92.2)
	Female	23 (7.5)
	Non binary	1 (0.3)
	Prefer not to answer	/
Level of education (*N *[%])	Graduate/Postgraduate	204 (47)
	High school	221 (51)
	Middle school	7 (2)
Employment status (*N *[%])	Full-time job	138 (31.9)
	Part-time job	36 (8.3)
	Unemployed	24 (5.6)
	Working student	111 (25.7)
	Student	123 (28.5)
Country of origin (*N *[%])	Italy	420 (97)
	Other European countries	6 (1.5)
	Non-European countries	6 (1.5)
Children (*N *[%])	Yes	38 (9)
	No	394 (91)
Vaginal deliveries (*N *[%]) (if children = yes)	Yes	30 (79)
	No	8 (21)
Current pregnancy (*N* [%])	Yes	3 (1)
	No	429 (99)
Gynecological conditions (*N* [%])	Yes	82 (19)
	No	351 (81)
Female genital self-image (*N* = 432)		
FGSIS-It (M ± SD)		20.4 ± 3.6
Sexuality (*N* = 264)		
FSFI (M ± SD)	Desire	3.8 ± 1.2
	Arousal	4.4 ± 1.7
	Lubrication	4.4 ± 1.7
	Orgasm	3.8 ± 1.8
	Satisfaction	4.6 ± 1.4
	Pain	4.4 ± 2.0
	FSFI-Total	25.3 ± 8.1
Psychological health (*N* = 413 for BAS-2 and PGWB-S; *N* = 373 for RSES)		
BAS-2 (M ± SD)		33.6 ± 7.2
PGWB-S (M ± SD)		22.0 ± 4.9
RSES (M ± SD)		18.5 ± 6.0

**Fig. 1:**
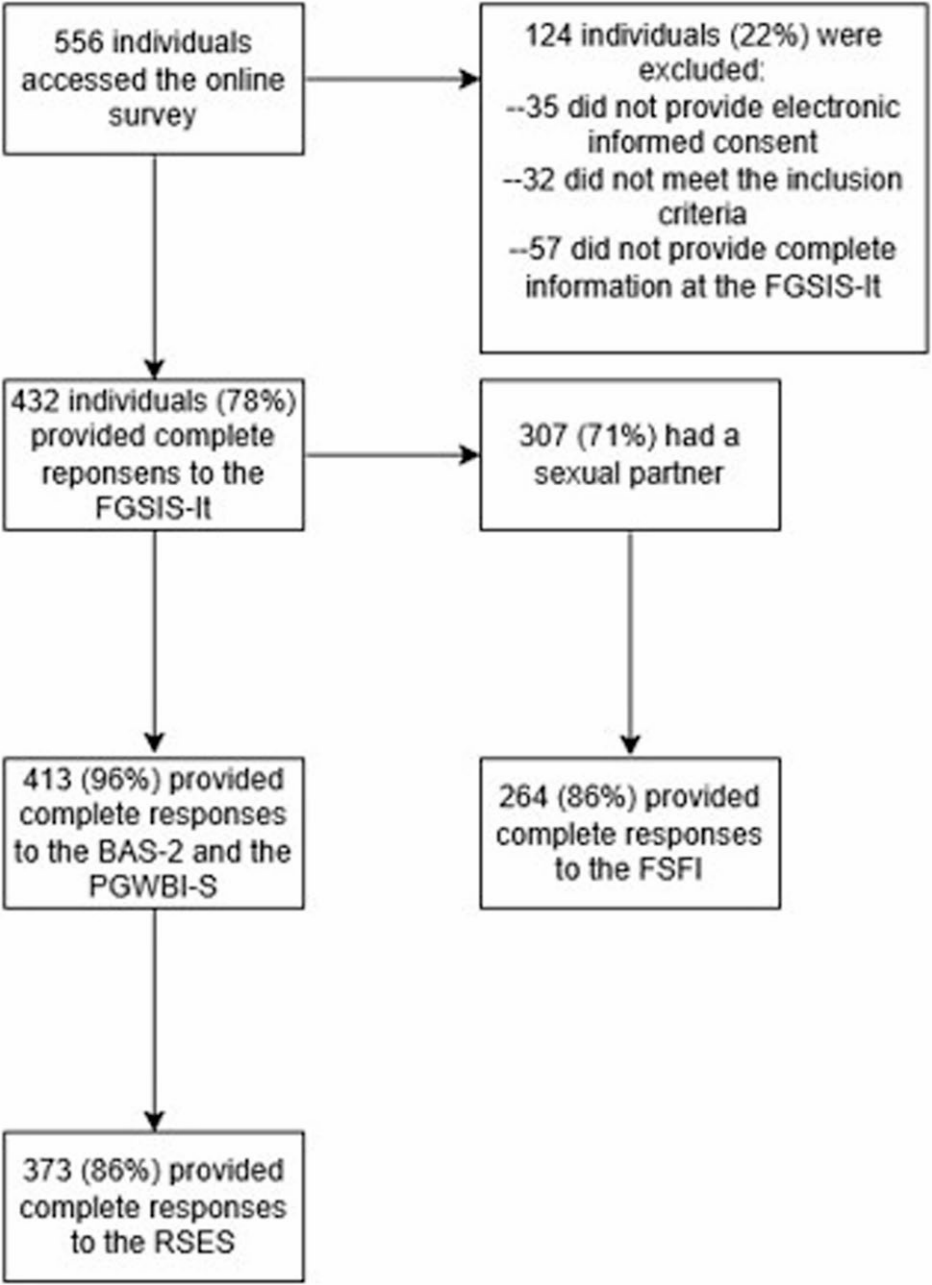
Participant flowchart

The online survey was accessed by 556 individuals. Of these, 35 did not provide electronic informed consent, and 32 did not meet the inclusion criteria and were automatically directed to the end of the survey. An additional 57 individuals abandoned the study before completing the FGSIS-It. The final sample was composed of 432 participants who provided complete responses to the FGSIS-It. A detailed flowchart is displayed in Fig. 1.

## Results

### Participant characteristics

The characteristics of the study sample are extensively reported in Table 1. Participants’ age ranged from 19 to 66 years (*M* = 26.14; *SD* = 9.26). The sample was mostly composed of relatively young, unmarried Italian individuals, with a sexual partner and without children, who were either employed or students. Among the minority of individuals who reported gynecological conditions, the most common diseases were PCOS (polycystic ovary syndrome), endometriosis with or without adenomyosis, HPV (human papillomavirus), and vulvodynia. This subgroup of participants reported slightly lower FGSIS-It global scores (*M* = 19.61; *SD* = 0.40) compared with individuals without gynecological conditions (*M* = 20.58; *SD* = 0.19), as clarified by a univariate analysis of variance (*F*[1,430] = 4.743, *p* = 0.030), although with a small effect size (partial *η²* = 0.011). An additional analysis of variance revealed no significant differences in FGSIS-It scores between participants who reported having had vaginal deliveries and those who did not (*p* = 0.627). The means and standard deviations of the questionnaires used to assess female sexual function and psychological health are also reported in Table 1. The score ranges were as follows: 2–36 for the FSFI, 11–50 for the BAS-2, 8–36 for the PGWB-S, and 1–30 for the RSES.

### The psychometric properties of the FGSIS-It

Overall, participants reported a relatively positive image of their external genitalia, with global FGSIS-It scores ranging from 10 to 28 (*M* = 20.40; *SD* = 3.60). As shown in Table 2 (left columns), the most highly endorsed item (based on the mean score) was “I feel positively about my genitals”, whereas Item 4 (“I think my genitals smell fine”) received the lowest score. The mean score for all items (except Item 1) was below 3 (“Agree” on the 4-point Likert scale), indicating that participants’ genital self-image was not entirely positive. Inter-item correlations within the FGSIS-It and correlations with the total score are reported in the right columns of Table 2. Pearson’s *r* values ranged from 0.685 (between Item 1 and Item 2) to 0.230 (between Item 5 and Item 7), indicating weaker, but still acceptable, inter-item association in light of the overall good internal consistency of the scale.

**Table 2:** FGSIS-It descriptive statistics and values of Pearson correlations (*N* = 432)

Item	M	SD	Median	Asymmetry	Kurtosis	1	2	3	4	5	6	7	FGSIS-It total
1. I feel positively about my genitals	3.16	0.675	3	-0.571	0.613		0.685^**^	0.485^**^	0.391^**^	0.432^**^	0.314^**^	0.517^**^	0.788^**^
2. I am satisfied with the appearance of my genitals	2.99	0.737	3	-0.363	-0.127			0.486^**^	0.350^**^	0.277^**^	0.306^**^	0.540^**^	0.755^**^
3. I would feel comfortable letting a sexual partner look at my genitals	2.91	0.768	3	-0.211	-0.495				0.318^**^	0.248^**^	0.284^**^	0.464^**^	0.688^**^
4. I think my genitals smell fine	2.74	0.670	3	-0.151	-0.064					0.309^**^	0.264^**^	0.295^**^	0.595^**^
5. I think my genitals work the way they are supposed to work	2.95	0.781	3	-0.418	-0.187						0.265^**^	0.230^**^	0.581^**^
6. I feel comfortable letting a healthcare provider examine my genitals	2.80	0.874	3	-0.252	-0.671							0.429^**^	0.626^**^
7. I am not embarrassed about my genitals	2.85	0.787	3	-0.243	-0.415								0.735^**^
FGSIS-It total	20.40	3.60	20	0.015	-0.231								

**The correlation is significant at the 0.01 level

EFA was conducted on the subsample 1, using the “eigenvalues greater than 1” criterion and excluding coefficients < 0.35, resulting in the extraction of a single factor that explained 41% of the variance. Bartlett’s test of sphericity was significant (*χ²* = 488.575, *p* < 0.001), and the Kaiser–Meyer–Olkin (KMO) measure of sampling adequacy was 0.798, indicating that the correlation matrix was suitable for factor analysis. Factor loadings ranged from 0.416 for Item 5 to 0.818 for Item 1 (see Table 3). The scale demonstrated good internal consistency, as indicated by Cronbach’s *α* (0.811) and McDonald’s *ω* values (0.823). Moreover, item–total correlations (see Table 2) were all substantial and statistically significant (ranging from 0.58 to 0.79), indicating that all items contribute meaningfully to the same latent construct.

**Table 3:** EFA factor loadings

Item	EFA Factor Loading (*N* = 201)
1. I feel positively about my genitals	0.818
2. I am satisfied with the appearance of my genitals	0.790
3. I would feel comfortable letting a sexual partner look at my genitals	0.662
4. I think my genitals smell fine	0.512
5. I think my genitals work the way they are supposed to work	0.416
6. I feel comfortable letting a healthcare provider examine my genitals	0.497
7. I am not embarrassed about my genitals	0.674

CFA was conducted on the second subgroup to compare the one-factor solution derived from EFA with the two-factor model described in the literature (factor 1: *Intrapersonal*; factor 2: *Interpersonal*). All CFA outputs are reported in detail in the supplementary material (see Supplemental Document 1). The adequacy of the one-factor model was supported, with standardized factor loadings ranging from 0.511 (Item 6) to 0.870 (Item 1). Most items showed moderate to strong loadings (e.g., Item 3 = 0.640; Item 7 = 0.750; Item 2 = 0.823; Item 1 = 0.870), indicating good item–factor relationships, while three items—Item 5 (0.544), Item 4 (0.532), and Item 6 (0.511)—had relatively moderate loadings.

Model fit indices indicated acceptable to good fit for the one-factor model. The chi-square test was non-significant (*χ²*(14) = 16.2, *p* = 0.302). All the considered indices supported model adequacy and met recommended thresholds for good fit: *CFI* = 0.999, *TLI* = 0.998, *SRMR* = 0.045, and *RMSEA* = 0.026 (90% CI = 0.000–0.071). *RMSEA* indicated close fit, although its upper confidence limit suggests a range of reasonable fit and should be interpreted with some caution. Modification indices did not indicate notable error covariances among items.

A two-factor model allowing a correlation between the latent factors was also tested. This model fit well (*CFI* = 1.000, *TLI* = 1.001, *SRMR* = 0.039; *RMSEA* = 0.000, 90% CI = 0.000–0.060, *p* = 0.891), but the factors were highly correlated (*r* = 0.899), suggesting substantial overlap. When the correlation was constrained to zero, model fit deteriorated dramatically (*CFI* = 0.522; *TLI* = 0.282; *SRMR* = 0.283; *RMSEA* = 0.526, 90% CI = 0.497–0.555, *p* < 0.001), indicating the factors could not be treated as independent. These results support the one-factor solution as the more parsimonious and theoretically appropriate representation of the data.

Table 4 presents the results of the Partial Credit Rasch Model (PCM) applied to the FGSIS-It scale. Item measures ranged from − 0.672 (Item 1) to 0.465 (Item 4), indicating a satisfactory spread of item difficulty along the latent continuum. Regarding item fit, Infit *MNSQ* values ranged from 0.667 to 1.267, and Outfit *MNSQ* values from 0.649 to 1.300, all within the acceptable range for the Rasch model. The distances between adjacent thresholds indicated adequate discrimination among response categories, with no evidence of disordered thresholds. The Person Separation Index (PSI) was 0.774, falling just below the conventional 0.80 threshold, yet still suggesting an acceptable level of reliability. Overall, the Rasch analysis supported the unidimensionality of the scale and confirmed the adequate fit of all items to the model.

**Table 4:** Results of the partial credit Rasch model (PCM)

FGSIS-It	Measure	S.E.Measure	Item fit		Thresholds		
			Infit	Outfit	1	2	3
Item 1	-0.672	0.09	0.667	0.649	-2.02	-0.92	2.94
Item 2	-0.231	0.09	0.794	0.792	-2.56	-0.39	2.95
Item 3	-0.076	0.09	0.992	1.014	-2.79	-0.08	2.87
Item 4	0.465	0.10	1.131	1.129	-3.53	-0.38	3.91
Item 5	-0.041	0.08	1.267	1.300	-2.27	-0.43	2.70
Item 6	0.377	0.08	1.249	1.264	-2.16	-0.14	2.30
Item 7	0.179	0.09	0.879	0.878	-2.59	-0.22	2.81

### Associations with sexual function and psychological health

The values of Pearson correlations between the FGSIS-It and the FSFI subscales and total score ranged between 0.217 for Orgasm to 0.317 for Desire, with all *p* values < 0.001 (see Table 4). To further explore associations between female genital self-image and risk of sexual dysfunction, we used ANOVA to compare the mean of the FGSIS-It in individuals younger than 45 years who reported having a current sexual partner (*N* = 246), categorized as at risk (*N* = 105; 43%) or not at risk (*N* = 141; 57%) of sexual dysfunction, controlling for the effects of potential confounders (age and presence of gynecological conditions). The analysis was statistically significant (*F* = 19.708, *p <* 0.001), with a medium effect size (partial *η²* = 0.075). Specifically, participants at risk of experiencing sexual dysfunction had a poorer genital self-image (*M* = 19.64; *SD* = 3.38) compared with participants not at risk (*M* = 21.63; *SD* = 3.22). The FGSIS-It also showed positive moderate-to-weak correlations with the BAS-2 (*r* = 0.404), the RSES (*r* = 0.315), and the PGWB-S (*r =* 0.188), with all *ps <* 0.001.

## Discussion

Fifteen years ago, Herbenick and Reece [2] developed and validated a 7-item instrument—the Female Genital Self-Image Scale (FGSIS)—to assess female genital self-image, which showed a positive, albeit weak, association with female sexual function. Since then, several translated versions have been validated, demonstrating not only the reliability of the FGSIS as a measurement tool, but also underscoring the relevance of the construct from psychological, sociocultural, and sexological perspectives [16–21]. However, until now, no validated Italian translation has been available.

Our study presents the psychometric properties of the first Italian translation of the FGSIS, referred to as the FGSIS-It. All items from the original scale were retained in the Italian version, which demonstrated good psychometric properties, evaluated through a rigorous statistical approach. First, we conducted an EFA, which revealed a one-factor solution, consistent with the original validation study [2] as well as other translation studies [18, 19, 21]. The CFA results confirmed the hypothesized unidimensional structure of the scale, which showed good internal consistency, with values of Cronbach’s *α* (0.811) and McDonald’s *ω* (0.823) comparable to those reported in the other validation studies. Overall, the psychometric properties of the scale appear adequate for continued use in research.

The sociodemographic characteristics of our sample were consistent with those of the original validation study by Herbenick and Reece [2], which included primarily young participants (mean age = 29.55 years) and was predominantly composed of heterosexual, sexually active individuals with a male sexual partner. The age range of participants was also comparable: 19–66 years in our study versus 18–68 years in the original study. Regarding the total FGSIS-It score, participants in our sample reported a relatively positive genital self-image (mean = 20.4), although this score was lower compared to findings from other studies [18, 20]. Our findings also suggest that individuals with gynecological conditions—including polycystic ovary syndrome (PCOS), endometriosis (a systemic rather than purely gynecological disease), human papillomavirus (HPV), and vulvodynia—tend to report lower female genital self-image. Interestingly, not all these conditions affect external genitalia. Although this issue was not systematically examined in the present study, and these conditions were only controlled for in our analyses, future research should more directly investigate female genital self-image in these populations. In this regard, evidence suggests that individuals with vulvar inflammatory dermatoses (e.g., lichen sclerosus, lichen planus, or vulvar dermatitis) report low levels of female genital self-image [40].

The second part of our statistical analyses, which focused on the associations between female genital self-image and both female sexual function and psychological health (including body appreciation, self-esteem, and psychological wellbeing), not only supports the validity of the scale—as shown in previous research [17]—but also highlights the importance of addressing female genital self-image in clinical settings. Consistent with previous research, including the original validation study [2], we found statistically significant positive correlations between female genital self-image and female sexual function, with correlation coefficients (*r*) ranging from weak to moderate. The novelty of our findings lies in the observed association between female genital self-image and risk of sexual dysfunction, as examined through ANOVA, which revealed a medium effect size.

The association between female genital self-image and sexual function was recently examined in a systematic review and meta-analysis by Alavi-Arjas et al. [41], who reported a positive moderate correlation (*r* = 0.375) between the two constructs, based on the findings of 16 studies. The causal direction of this association remains unclear. Alavi-Arjas et al. [41] hypothesized a bidirectional relationship between genital self-image and sexual function, suggesting that the two constructs may influence each other through a reciprocal interaction. In a study by Soares and colleagues [42], higher scores on the FGSIS were associated with increased scores in the satisfaction, arousal, and orgasm domains of the FSFI. Women who viewed masturbation positively—reporting feelings of empowerment and satisfaction—also tended to have higher FSFI scores. In contrast, those experiencing negative emotions such as shame or guilt during masturbation showed significantly lower scores on both the FSFI and FGSIS. Overall, research suggests that body and genital self-image have a direct impact on female sexual function, which emerges from a complex interplay of biopsychosocial and cultural factors [3]. Based on these findings, the positive moderate correlations between female genital self-image and both body appreciation and self-esteem revealed by our analyses are not surprising and confirm the convergent validity of the scale. The correlation between the FGSIS-It and the PGWB-S was significantly positive but weak. We acknowledge that these analyses did not directly address discriminant validity, i.e., the extent to which the FGSIS-It measures a construct distinct from related constructs such as general self-esteem. However, the correlations ranged from weak to moderate, suggesting that the FGSIS-It is related to these constructs while still capturing a specific and distinct dimension.

In summary, our study provides a unique contribution to existing research and clinical practice by offering the first validated Italian translation of the FGSIS. The questionnaire has been shown to be reliable and psychometrically sound for measuring this key construct, which is also known to influence individuals’ decisions to undergo female genital cosmetic surgery, particularly among younger populations [7, 43]. In this context, our findings underscore the importance of considering female genital self-image when assessing female sexual function and psychological wellbeing, especially in relation to body image and self-esteem.

The anonymous, online approach to data collection employed in our study is particularly suitable for gathering information on such sensitive topics. However, the study design did not permit an assessment of the scale’s test–retest reliability, which constitutes the primary limitation of our research. When data are collected online, researchers have less control over participant selection, which may introduce self-selection bias. In this specific case, individuals with greater concerns related to female genital self-image may have been more likely to participate, potentially contributing to the lower overall FGSIS-It score observed compared to other studies. The use of an educational platform (Blackboard Learn) may also have “attracted” more educated and sexually health-conscious participants. The lack of quantitative data on female genital self-image in Italy did not allow for comparisons with the general population. Moreover, our study population—although similar to those in other studies, including the validation study of the original English version of the FGSIS [2]—was homogeneous, as the sample primarily consisted of young, heterosexual, and sexually active individuals. For this reason, comparative analyses between key subgroups were not feasible.

Future studies should investigate the test–retest reliability of the FGSIS-It and examine its psychometric properties in clinical populations, including individuals with gynecological conditions. The role of female genital self-image remains largely overlooked in Italy. Our study provides a validated tool to gain deeper insight into how Italian individuals assigned female at birth perceive their genitals and how this perception may be associated with health-related behaviors—such as participation in screening programs (e.g., for HPV)—and decisions regarding female genital cosmetic surgery. Furthermore, our findings highlight the need for additional research to understand female genital self-image from a biopsychosocial perspective, addressing the complex interplay between body, mind, and culture.

## Conclusion

In our study, the FGSIS-It showed good psychometric properties in terms of validity and reliability and appeared to be an adequate instrument for assessing how Italian individuals assigned female at birth perceive their genitals. Although further research is needed to evaluate its test–retest reliability, this study represents an important starting point, offering the first Italian validation of the FGSIS originally developed by Herbenick and Reece [2].

## Supplementary Information

Supplementary Material 1.

Supplementary Material 2.

Supplementary Material 3.

## Data Availability

The data supporting this article will be shared upon reasonable request to the corresponding author.

## Abbreviations

BAS-2: Body Appreciation Scale-2
CFA: Confirmatory Factor Analysis
EFA: Exploratory Factor Analysis
FGSIS: Female Genital Self-Image Scale
FGSIS-It: Female Genital Self-Image Scale-Italian
FSIS: Female Sexual Function Index
HPV: Human papillamovirus
KMO: Kaiser–Meyer–Olkin
PCOS: Polycystic Ovary Syndrome
PGWB-S: Psychological General Wellbeing Index-Short
RSES: Rosenberg Self-Esteem Scale
