# Supplementary material for: The Italian version of the Female Genital Self-Image Scale: psychometric properties and associations with sexual function and psychological health
Source: BMC Psychol. 2026 Jan 28;14:252. doi: 10.1186/s40359-026-04030-6 (PMC12922398; doi:10.1186/s40359-026-04030-6)
Supplement: Supplementary file 1 — Supplementary Material 1. [file 40359_2026_4030_MOESM1_ESM.docx]

**Supplemental Table 2.** The original version of the FGSIS and the FGSIS-It

| **Original FGSIS** | **FGSIS-It^*^** |
| --- | --- |
| 1. I feel positively about my genitals | 1. Mi sento bene rispetto ai miei genitali |
| 2. I am satisfied with the appearance of my genitals | 2. Sono soddisfatt*** di come i miei genitali appaiono |
| 3. I would feel comfortable letting a sexual partner look at my genitals | 3. Mi sentirei a mio agio nel lasciare che un*** partner sessuale guardi i miei genitali |
| 4. I think my genitals smell fine | 4. Penso che i miei genitali abbiano un buon odore |
| 5. I think my genitals work the way they are supposed to work | 5. Penso che i miei genitali funzionino come dovrebbero |
| 6. I feel comfortable letting a healthcare provider examine my genitals | 6. Mi sento a mio agio nel lasciare che un operatore sanitario esamini i miei genitali |
| 7. I am not embarrassed about my genitals | 7. Non sono imbarazzat*** per i miei genitali |
| Answers:   1. Strongly disagree 2. Disagree 3. Agree 4. Strongly agree | Risposte:   1. Fortemente in disaccordo 2. In disaccordo 3. D’accordo 4. Fortemente d’accordo |

^*^ Italian grammar requires nouns and adjectives to be gendered (masculine or feminine). The FGSIS-It was developed using inclusive language, as our study included individuals assigned female at birth, regardless of their gender identity. In some cases, we employed the asterisk symbol (*) to avoid gendered endings. However, for simplicity, “healthcare provider” was translated using a generic masculine form (“operatore sanitario”), which in Italian is conventionally understood as gender-neutral when referring to professional roles or functions.
