# Supplementary material for: The Italian version of the Female Genital Self-Image Scale: psychometric properties and associations with sexual function and psychological health
Source: BMC Psychol. 2026 Jan 28;14:252. doi: 10.1186/s40359-026-04030-6 (PMC12922398; doi:10.1186/s40359-026-04030-6)
Supplement: Supplementary file 2 — Supplementary Material 2. [file 40359_2026_4030_MOESM2_ESM.docx]

**Supplemental Table 1.** Summary of the Female Genital-Self Image Scale (FGSIS) validation studies (original version and available translations)

| **Authors** | **Language** | **EFA or PCA** | **CFA** | **Factorial solution** | **FGSIS version** | **Test-retest reliability** |
| --- | --- | --- | --- | --- | --- | --- |
| Herbenick and Reece, 2010 | English (USA) | Yes | No | One factor | 7-item | No |
| Herbenick et al., 2011 | English (USA) | No | Yes | One factor | Good fit for both the 4-item and the 7-item version | Yes |
| DeMaria et al., 2012 | English (USA) | Yes | Yes | Two factors | 7-item | No |
| Mohammed and Hassan, 2014 | Arabic | No | No | Not verified | 4-item | Yes |
| Pakpour et al., 2014 | Persian | Yes | Yes | Two factors | 7-item | Yes |
| Kaya et al., 2019 | Turkish | Yes | Yes | One factor (EFA)  Two factors (CFA) | 7-item | Yes |
| Bartolomé et al., 2022 | Spanish | Yes | No | One factor | 7-item | Yes |
| Komon et al., 2022 | Thai | No | Yes | Two factors | 7-item | Yes |
| Loewinski et al., 2022 | Swedish | No | No | Not verified | 7-item | No |

*Abbreviations:*

CFA: Confirmatory Factor Analysis

EFA: Exploratory Factor Analysis

PCA: Principal Component Analysis
