# Supplementary material for: The Italian version of the Female Genital Self-Image Scale: psychometric properties and associations with sexual function and psychological health
Source: BMC Psychol. 2026 Jan 28;14:252. doi: 10.1186/s40359-026-04030-6 (PMC12922398; doi:10.1186/s40359-026-04030-6)

Confirmatory Factor Analysis (CFA)

One-factor model

Models Info

**Estimation Method**	**DWLS**
**Optimization Method**	**NLMINB**
**Number of observations**	**231**
**Free parameters**	**28**
**Standard errors**	**Robust**
**Scaled test**	**None**
**Converged**	**TRUE**
**Iterations**	**18**
**Model**	**Endogenous1=~FGSIS_1+FGSIS_2+FGSIS_3+FGSIS_4+FGSIS_5+FGSIS_6+FGSIS_7**

Nota. Variable (FGSIS_1,FGSIS_2,FGSIS_3,FGSIS_4,FGSIS_5,FGSIS_6,FGSIS_7) has been coerced to ordered type.

Overall Tests

Model tests

Label	X^2	df	p
**User Model**	**16.2**	**14**	**0.302**
Baseline Model	1880.7	21	<.001

Fit indices

		90% Confidence Intervals		
SRMR	RMSEA	Lower	Upper	RMSEA p
**0.045**	**0.026**	**0.000**	**0.071**	**0.763**

User model versus baseline model

	Model
**Comparative Fit Index (CFI)**	**0.999**
**Tucker-Lewis Index (TLI)**	**0.998**
**Bentler-Bonett Non-normed Fit Index (NNFI)**	0.998
**Relative Noncentrality Index (RNI)**	0.999
**Bentler-Bonett Normed Fit Index (NFI)**	0.991
**Bollen's Relative Fit Index (RFI)**	0.987
**Bollen's Incremental Fit Index (IFI)**	0.999
**Parsimony Normed Fit Index (PNFI)**	0.661

Estimates

Measurement model

				90% Confidence Intervals				
**Latent**	**Observed**	**Estimate**	**SE**	**Lower**	**Upper**	**β**	**z**	**p**
**Endogenous1**	**FGSIS_1**	1.000	0.0000	1.000	1.000	**0.870**		
	**FGSIS_2**	0.946	0.0404	0.879	1.012	**0.823**	23.40	<.001
	**FGSIS_3**	0.736	0.0499	0.654	0.818	**0.640**	14.75	<.001
	**FGSIS_4**	0.611	0.0695	0.497	0.726	**0.532**	8.79	<.001
	**FGSIS_5**	0.625	0.0608	0.525	0.725	**0.544**	10.29	<.001
	**FGSIS_6**	0.588	0.0622	0.486	0.690	**0.511**	9.45	<.001
	**FGSIS_7**	0.862	0.0471	0.784	0.940	**0.750**	18.28	<.001

Variances and Covariances

Variable 1	Variable 2	Estimate	SE	90% Confidence Intervals		β	z	p
				Lower	Upper			
FGSIS_1	FGSIS_1	0.243	0.0000	0.243	0.243	0.243		
FGSIS_2	FGSIS_2	0.323	0.0000	0.323	0.323	0.323		
FGSIS_3	FGSIS_3	0.590	0.0000	0.590	0.590	0.590		
FGSIS_4	FGSIS_4	0.717	0.0000	0.717	0.717	0.717		
FGSIS_5	FGSIS_5	0.704	0.0000	0.704	0.704	0.704		
FGSIS_6	FGSIS_6	0.738	0.0000	0.738	0.738	0.738		
FGSIS_7	FGSIS_7	0.438	0.0000	0.438	0.438	0.438		
Endogenous1	Endogenous1	0.757	0.0458	0.681	0.832	1.000	16.5	<.001

Intercepts

Variable	Intercept	SE	90% Confidence Intervals		z	p
			Lower	Upper		
FGSIS_1	0.000	0.000	0.000	0.000		
FGSIS_2	0.000	0.000	0.000	0.000		
FGSIS_3	0.000	0.000	0.000	0.000		
FGSIS_4	0.000	0.000	0.000	0.000		
FGSIS_5	0.000	0.000	0.000	0.000		
FGSIS_6	0.000	0.000	0.000	0.000		
FGSIS_7	0.000	0.000	0.000	0.000		
Endogenous1	0.000	0.000	0.000	0.000		

Thresholds

Variable	Step	Thresholds	SE	90% Confidence Intervals		z	p
				Lower	Upper		
FGSIS_1	t1	-0.553	0.087	-0.697	-0.409	-6.330	<.001
FGSIS_1	t2	1.068	0.102	0.899	1.236	10.440	<.001
FGSIS_1	t3	2.021	0.185	1.716	2.326	10.903	<.001
FGSIS_2	t1	-0.712	0.091	-0.862	-0.563	-7.852	<.001
FGSIS_2	t2	0.755	0.092	0.604	0.906	8.225	<.001
FGSIS_2	t3	1.944	0.174	1.658	2.229	11.183	<.001
FGSIS_3	t1	-0.814	0.093	-0.968	-0.660	-8.716	<.001

FGSIS_3	t2	0.631	0.089	0.485	0.777	7.096	<.001
FGSIS_3	t3	1.944	0.174	1.658	2.229	11.183	<.001
FGSIS_4	t1	-1.362	0.118	-1.555	-1.169	-11.589	<.001
FGSIS_4	t2	0.372	0.085	0.233	0.511	4.388	<.001
FGSIS_4	t3	1.763	0.151	1.514	2.012	11.654	<.001
FGSIS_5	t1	-0.658	0.089	-0.805	-0.510	-7.349	<.001
FGSIS_5	t2	0.671	0.090	0.523	0.819	7.475	<.001
FGSIS_5	t3	1.714	0.146	1.473	1.954	11.733	<.001
FGSIS_6	t1	-0.876	0.095	-1.033	-0.719	-9.197	<.001
FGSIS_6	t2	0.269	0.084	0.131	0.407	3.213	0.001
FGSIS_6	t3	1.362	0.118	1.169	1.555	11.589	<.001
FGSIS_7	t1	-0.925	0.097	-1.084	-0.766	-9.549	<.001
FGSIS_7	t2	0.467	0.086	0.325	0.608	5.427	<.001
FGSIS_7	t3	1.587	0.134	1.366	1.807	11.829	<.001

Two-factor model with correlated factors

Models Info

**Estimation Method**	**DWLS**	.
**Optimization Method**	**NLMINB**	
**Number of observations**	**231**	
**Free parameters**	**29**	
**Standard errors**	**Robust**	
**Scaled test**	**None**	
**Converged**	**TRUE**	
**Iterations**	**22**	
**Model**	**Endogenous1=~FGSIS_1+FGSIS_2+FGSIS_4+FGSIS_5**	
	**Endogenous2=~FGSIS_6+FGSIS_7+FGSIS_3**	

Nota. Variable (FGSIS_1,FGSIS_2,FGSIS_4,FGSIS_5,FGSIS_6,FGSIS_7,FGSIS_3) has been coerced to ordered type.

Overall Tests

Model tests

Label	X^2	df	p
**User Model**	**11.9**	**13**	**0.539**
Baseline Model	1880.7	21	<.001

Fit indices

		90% Confidence Intervals		
SRMR	RMSEA	Lower	Upper	RMSEA p
**0.039**	**0.000**	**0.000**	**0.060**	**0.891**

User model versus baseline model

	Model
**Comparative Fit Index (CFI)**	**1.000**
**Tucker-Lewis Index (TLI)**	**1.001**
**Bentler-Bonett Non-normed Fit Index (NNFI)**	1.001
**Relative Noncentrality Index (RNI)**	1.001
**Bentler-Bonett Normed Fit Index (NFI)**	0.994
**Bollen's Relative Fit Index (RFI)**	0.990
**Bollen's Incremental Fit Index (IFI)**	1.001
**Parsimony Normed Fit Index (PNFI)**	0.615

Estimates

Measurement model

				90% Confidence Intervals				
Latent	Observed	Estimate	SE	Lower	Upper	β	z	p
**Endogenous1**	**FGSIS_1**	1.000	0.0000	1.000	1.000	**0.883**		
	**FGSIS_2**	0.943	0.0414	0.875	1.011	**0.833**	22.80	<.001

	**FGSIS_4**	0.611	0.0693	0.497	0.725	**0.540**	8.81	<.001
	**FGSIS_5**	0.626	0.0608	0.526	0.726	**0.553**	10.29	<.001
**Endogenous2**	**FGSIS_6**	1.000	0.0000	1.000	1.000	**0.532**		
	**FGSIS_7**	1.496	0.1599	1.233	1.759	**0.796**	9.36	<.001
	**FGSIS_3**	1.252	0.1489	1.007	1.496	**0.666**	8.41	<.001

Variances and Covariances

				90% Confidence Intervals				
**Variable 1**	**Variable 2**	**Estimate**	**SE**	**Lower**	**Upper**	**β**	**z**	**p**
FGSIS_1	FGSIS_1	0.220	0.0000	0.220	0.220	0.220		
FGSIS_2	FGSIS_2	0.306	0.0000	0.306	0.306	0.306		
FGSIS_4	FGSIS_4	0.709	0.0000	0.709	0.709	0.709		
FGSIS_5	FGSIS_5	0.694	0.0000	0.694	0.694	0.694		
FGSIS_6	FGSIS_6	0.717	0.0000	0.717	0.717	0.717		
FGSIS_7	FGSIS_7	0.366	0.0000	0.366	0.366	0.366		
FGSIS_3	FGSIS_3	0.556	0.0000	0.556	0.556	0.556		
Endogenous1	Endogenous1	0.780	0.0477	0.702	0.859	1.000	16.34	<.001
Endogenous2	Endogenous2	0.283	0.0578	0.188	0.378	1.000	4.90	<.001
Endogenous1	Endogenous2	0.422	0.0487	0.342	0.502	0.899	8.67	<.001

Intercepts

			90% Confidence Intervals			
**Variable**	**Intercept**	**SE**	**Lower**	**Upper**	**z**	**p**
FGSIS_1	0.000	0.000	0.000	0.000		

FGSIS_2	0.000	0.000	0.000	0.000
FGSIS_4	0.000	0.000	0.000	0.000
FGSIS_5	0.000	0.000	0.000	0.000
FGSIS_6	0.000	0.000	0.000	0.000
FGSIS_7	0.000	0.000	0.000	0.000
FGSIS_3	0.000	0.000	0.000	0.000
Endogenous1	0.000	0.000	0.000	0.000
Endogenous2	0.000	0.000	0.000	0.000

Thresholds

				90% Confidence Intervals			
**Variable**	**Step**	**Thresholds**	**SE**	**Lower**	**Upper**	**z**	**p**
FGSIS_1	t1	-0.553	0.087	-0.697	-0.409	-6.330	<.001
FGSIS_1	t2	1.068	0.102	0.899	1.236	10.440	<.001
FGSIS_1	t3	2.021	0.185	1.716	2.326	10.903	<.001
FGSIS_2	t1	-0.712	0.091	-0.862	-0.563	-7.852	<.001
FGSIS_2	t2	0.755	0.092	0.604	0.906	8.225	<.001
FGSIS_2	t3	1.944	0.174	1.658	2.229	11.183	<.001
FGSIS_4	t1	-1.362	0.118	-1.555	-1.169	-11.589	<.001
FGSIS_4	t2	0.372	0.085	0.233	0.511	4.388	<.001
FGSIS_4	t3	1.763	0.151	1.514	2.012	11.654	<.001
FGSIS_5	t1	-0.658	0.089	-0.805	-0.510	-7.349	<.001
FGSIS_5	t2	0.671	0.090	0.523	0.819	7.475	<.001
FGSIS_5	t3	1.714	0.146	1.473	1.954	11.733	<.001
FGSIS_6	t1	-0.876	0.095	-1.033	-0.719	-9.197	<.001

FGSIS_6	t2	0.269	0.084	0.131	0.407	3.213	0.001
FGSIS_6	t3	1.362	0.118	1.169	1.555	11.589	<.001
FGSIS_7	t1	-0.925	0.097	-1.084	-0.766	-9.549	<.001
FGSIS_7	t2	0.467	0.086	0.325	0.608	5.427	<.001
FGSIS_7	t3	1.587	0.134	1.366	1.807	11.829	<.001
FGSIS_3	t1	-0.814	0.093	-0.968	-0.660	-8.716	<.001
FGSIS_3	t2	0.631	0.089	0.485	0.777	7.096	<.001
FGSIS_3	t3	1.944	0.174	1.658	2.229	11.183	<.001

Additional outputs

Reliability indices

Variable	α	Ordinal α	ω_1	ω_2	ω_3	AVE
Endogenous1	0.733	0.796	0.738	0.738	0.730	0.518
Endogenous2	0.630	0.699	0.643	0.643	0.641	0.454

Path Model

Path diagrams

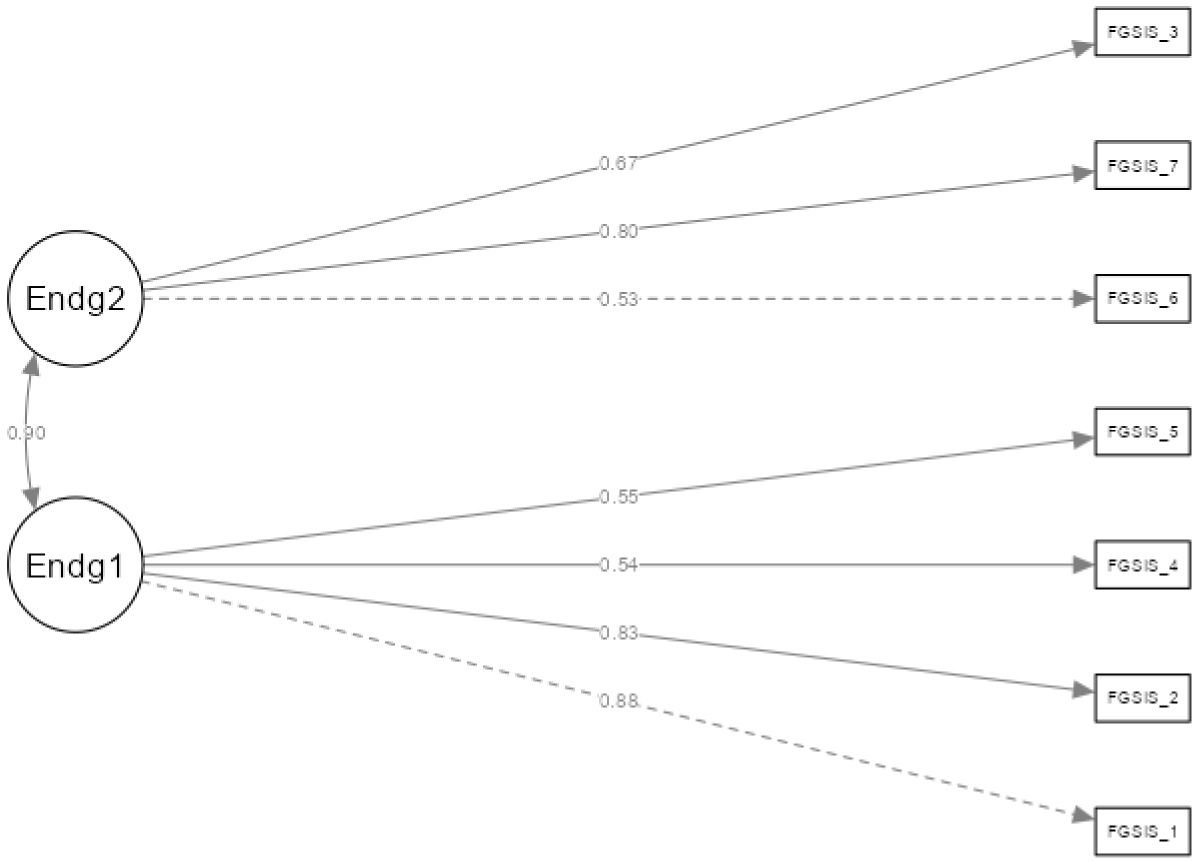

Two-factor model with uncorrelated factors

Models Info

**Estimation Method**	**DWLS**
**Optimization Method**	**NLMINB**
**Number of observations**	**231**
**Free parameters**	**28**
**Standard errors**	**Robust**
**Scaled test**	**None**
**Converged**	**TRUE**
**Iterations**	**20**
**Model**	**Endogenous1=~FGSIS_1+FGSIS_2+FGSIS_4+FGSIS_5**
	**Endogenous2=~FGSIS_6+FGSIS_7+FGSIS_3**

Nota. Variable (FGSIS_1,FGSIS_2,FGSIS_4,FGSIS_5,FGSIS_6,FGSIS_7,FGSIS_3) has been coerced to ordered type.

Overall Tests

Model tests

Label	X^2	df	p
**User Model**	**904**	**14**	**<.001**
Baseline Model	1881	21	<.001

Fit indices

		90% Confidence Intervals		
SRMR	RMSEA	Lower	Upper	RMSEA p
**0.283**	**0.526**	**0.497**	**0.555**	**<.001**

User model versus baseline model

	Model
**Comparative Fit Index (CFI)**	**0.522**
**Tucker-Lewis Index (TLI)**	**0.282**
**Bentler-Bonett Non-normed Fit Index (NNFI)**	0.282
**Relative Noncentrality Index (RNI)**	0.522
**Bentler-Bonett Normed Fit Index (NFI)**	0.520
**Bollen's Relative Fit Index (RFI)**	0.279
**Bollen's Incremental Fit Index (IFI)**	0.523
**Parsimony Normed Fit Index (PNFI)**	0.346

Estimates

Measurement model

				90% Confidence Intervals				
Latent	Observed	Estimate	SE	Lower	Upper	β	z	p
**Endogenous1**	**FGSIS_1**	1.000	0.0000	1.000	1.000	**0.929**		
	**FGSIS_2**	0.834	0.0801	0.702	0.965	**0.774**	10.40	<.001

	**FGSIS_4**	0.592	0.0717	0.474	0.710	**0.549**	8.25	<.001
	**FGSIS_5**	0.632	0.0701	0.517	0.748	**0.587**	9.02	<.001
**Endogenous2**	**FGSIS_6**	1.000	0.0000	1.000	1.000	**0.552**		
	**FGSIS_7**	1.564	0.2995	1.072	2.057	**0.864**	5.22	<.001
	**FGSIS_3**	1.065	0.1201	0.867	1.262	**0.588**	8.87	<.001

Variances and Covariances

				90% Confidence Intervals				
**Variable 1**	**Variable 2**	**Estimate**	**SE**	**Lower**	**Upper**	**β**	**z**	**p**
FGSIS_1	FGSIS_1	0.138	0.0000	0.138	0.138	0.138		
FGSIS_2	FGSIS_2	0.401	0.0000	0.401	0.401	0.401		
FGSIS_4	FGSIS_4	0.698	0.0000	0.698	0.698	0.698		
FGSIS_5	FGSIS_5	0.655	0.0000	0.655	0.655	0.655		
FGSIS_6	FGSIS_6	0.695	0.0000	0.695	0.695	0.695		
FGSIS_7	FGSIS_7	0.254	0.0000	0.254	0.254	0.254		
FGSIS_3	FGSIS_3	0.654	0.0000	0.654	0.654	0.654		
Endogenous1	Endogenous1	0.862	0.0807	0.729	0.995	1.000	10.68	<.001
Endogenous2	Endogenous2	0.305	0.0720	0.186	0.423	1.000	4.24	<.001
Endogenous1	Endogenous2	0.000	0.0000	0.000	0.000	0.000		

Intercepts

			90% Confidence Intervals			
**Variable**	**Intercept**	**SE**	**Lower**	**Upper**	**z**	**p**
FGSIS_1	0.000	0.000	0.000	0.000		

FGSIS_2	0.000	0.000	0.000	0.000
FGSIS_4	0.000	0.000	0.000	0.000
FGSIS_5	0.000	0.000	0.000	0.000
FGSIS_6	0.000	0.000	0.000	0.000
FGSIS_7	0.000	0.000	0.000	0.000
FGSIS_3	0.000	0.000	0.000	0.000
Endogenous1	0.000	0.000	0.000	0.000
Endogenous2	0.000	0.000	0.000	0.000

Thresholds

				90% Confidence Intervals			
**Variable**	**Step**	**Thresholds**	**SE**	**Lower**	**Upper**	**z**	**p**
FGSIS_1	t1	-0.553	0.087	-0.697	-0.409	-6.330	<.001
FGSIS_1	t2	1.068	0.102	0.899	1.236	10.440	<.001
FGSIS_1	t3	2.021	0.185	1.716	2.326	10.903	<.001
FGSIS_2	t1	-0.712	0.091	-0.862	-0.563	-7.852	<.001
FGSIS_2	t2	0.755	0.092	0.604	0.906	8.225	<.001
FGSIS_2	t3	1.944	0.174	1.658	2.229	11.183	<.001
FGSIS_4	t1	-1.362	0.118	-1.555	-1.169	-11.589	<.001
FGSIS_4	t2	0.372	0.085	0.233	0.511	4.388	<.001
FGSIS_4	t3	1.763	0.151	1.514	2.012	11.654	<.001
FGSIS_5	t1	-0.658	0.089	-0.805	-0.510	-7.349	<.001
FGSIS_5	t2	0.671	0.090	0.523	0.819	7.475	<.001
FGSIS_5	t3	1.714	0.146	1.473	1.954	11.733	<.001
FGSIS_6	t1	-0.876	0.095	-1.033	-0.719	-9.197	<.001

FGSIS_6	t2	0.269	0.084	0.131	0.407	3.213	0.001
FGSIS_6	t3	1.362	0.118	1.169	1.555	11.589	<.001
FGSIS_7	t1	-0.925	0.097	-1.084	-0.766	-9.549	<.001
FGSIS_7	t2	0.467	0.086	0.325	0.608	5.427	<.001
FGSIS_7	t3	1.587	0.134	1.366	1.807	11.829	<.001
FGSIS_3	t1	-0.814	0.093	-0.968	-0.660	-8.716	<.001
FGSIS_3	t2	0.631	0.089	0.485	0.777	7.096	<.001
FGSIS_3	t3	1.944	0.174	1.658	2.229	11.183	<.001

Additional outputs

Reliability indices

Variable	α	Ordinal α	ω_1	ω_2	ω_3	AVE
Endogenous1	0.733	0.796	0.746	0.746	0.747	0.527
Endogenous2	0.630	0.699	0.653	0.653	0.653	0.465

Path Model

Path diagrams

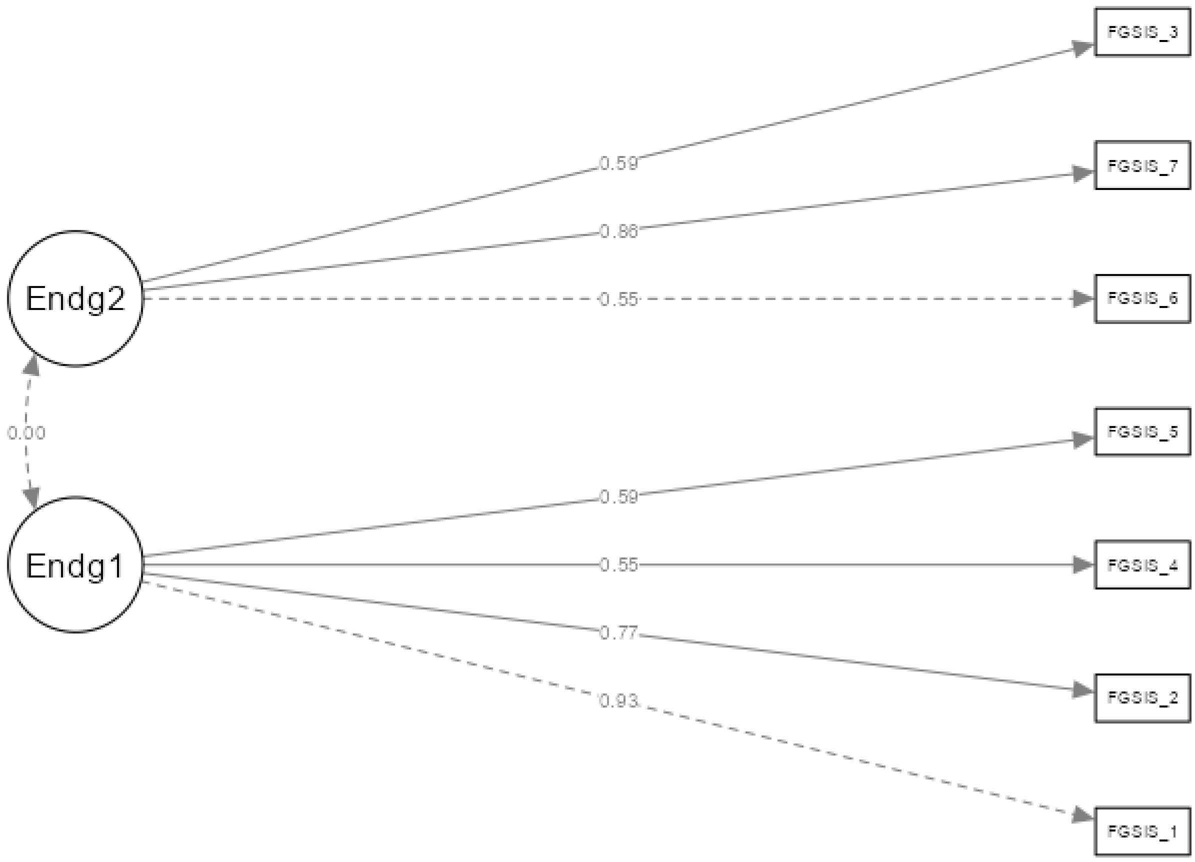

Supplement: Supplementary file 3 — Supplementary Material 3. [file 40359_2026_4030_MOESM3_ESM.pdf]
